# Association between triglyceride-glucose-atherogenic index of plasma and cardiovascular disease in middle-aged and older Chinese and American individuals: A cross-sectional analysis of two nationwide cohort datasets

**DOI:** 10.1097/MD.0000000000048675

**Published:** 2026-05-08

**Authors:** Yuanfeng Wu, Yingni Chen, Qiang Wu, Chulin Wang, Xianxuan Wang

**Affiliations:** a Department of Cardiology, Jieyang People’s Hospital, Jieyang, China; b Department of Finance, Jieyang People’s Hospital, Jieyang, China.

**Keywords:** atherogenic index of plasma, cardiovascular disease, CHARLS, NHANES, triglyceride-glucose index

## Abstract

The relationship between the triglyceride-glucose (TyG) index combined with the atherogenic index of plasma (AIP) and cardiovascular disease (CVD) remains unclear in both Chinese and American populations. This analytic cross-sectional study used data from two nationwide datasets: 10,342 participants from the China Health and Retirement Longitudinal Study and 4185 from the National Health and Nutrition Examination Survey. Multivariable logistic regression, restricted cubic spline analysis, and receiver operating characteristic curve analysis were used to investigate the relationship between TyG, AIP, TyG-AIP, and CVD. The results showed a significant positive association between high levels of TyG index, AIP, and TyG-AIP with the risk of CVD. The estimates were 1.66 (95% CI: 1.41–1.95) and 1.84 (95% CI: 1.43–2.37) for TyG index, 1.69 (95% CI: 1.43–1.99) and 1.97 (95% CI: 1.53–2.54) for AIP, and 1.68 (95% CI: 1.43–1.98) and 1.97 (95% CI: 1.53–2.54) for TyG-AIP in China health and retirement longitudinal study and National Health and Nutrition Examination Survey, respectively. Restricted cubic spline analyses showed that most of the above relationships were linear (*P*-overall < .001, *P*-nonlinear > .05). Receiver operating characteristic analysis showed that TyG-AIP had better discriminatory ability for CVD than TyG or AIP alone. The study demonstrated linear associations between higher TyG index, AIP, and TyG-AIP levels and increased CVD risk in middle-aged and older populations. Of the indicators evaluated, TyG-AIP showed the greatest ability to distinguish individuals with CVD.

## 1. Introduction

Cardiovascular disease (CVD) remains the leading cause of death and disability worldwide. In 2019, nearly 19.8 million deaths were attributed to CVD.^[1]^ The number of patients living with CVD has also increased over time, rising by around 252 million between 1990 and 2019.^[2]^ This growing number of patients has created a heavy burden on healthcare systems and society. For example, the estimated annual cost related to CVD in the United States reached nearly $378 billion during 2017–2018.^[3]^ Therefore, identifying modifiable risk factors is important for improving early prevention and reducing the future burden of CVD.

Previous studies have reported that insulin resistance is an important risk factor for CVD. Insulin resistance has been associated with hypertension, diabetes, arterial stiffness, and obesity, and is considered an independent risk factor for CVD events.^[4,5]^ Therefore, early identification of insulin resistance can help prevent or delay the progression of CVD. The triglyceride-glucose index (TyG) is calculated from fasting triglyceride (TG) and fasting blood glucose (FBG) levels and is a reliable and sensitive marker of insulin resistance and has demonstrated an independent risk factor for cardiovascular outcomes.^[6–10]^ Long-term exposure to elevated TyG has also been linked to poor prognosis in patients with CVD.^[11,12]^ Another common metabolic problem is atherogenic dyslipidemia. Atherogenic dyslipidemia is common in individuals with obesity, metabolic syndrome, and type 2 diabetes mellitus. It is characterized by high triglyceride levels and low high-density lipoprotein cholesterol.^[13–16]^ The atherogenic index of plasma (AIP) is expressed as log10 (TG/high-density lipoprotein cholesterol [HDL-C]) and is considered a useful biomarker for assessing CVD risk assessment.^[17–19]^

Recent studies have combined the TyG index with obesity-related indicators, such as TyG-BMI, the triglyceride-glucose index combined with waist circumference, and the triglyceride-glucose index combined with waist-to-height ratio (TyG-WHtR). These combined indices were associated with a higher risk of CVD than single indicators in previous studies.^[20–22]^ Some population-based studies also found that individuals with both a high TyG index and high AIP had a higher risk of coronary artery disease, especially among patients with acute coronary syndrome.^[23]^ The triglyceride-glucose index, combined with the atherogenic index of plasma (TyG-AIP) combines the TyG index with AIP and includes information from both glucose and lipid metabolism.^[24]^ However, studies on TyG-AIP and CVD are still limited. This is especially true in middle-aged and older adults from different populations. Moreover, few studies have focused on the dose–response relationship between these indices and CVD. In this study, we used data from the China Health and Retirement Longitudinal Study (CHARLS) and the National Health and Nutrition Examination Survey (NHANES) to examine the association between TyG-AIP and CVD.

## 2. Method

### 2.1. Study population

This was an analytic cross-sectional study based on two nationally representative datasets, CHARLS from China and NHANES from the United States. The inclusion of these two populations enabled assessment of the association between TyG-related indices and CVD across different ethnic, socioeconomic, and healthcare contexts.

A detailed description of CHARLS and NHANES has been published previously.^[25,26]^ CHARLS is a nationwide survey of adults aged 45 years and older in China and has been conducted every 2 to 3 years. In contrast, NHANES is a nationally representative cross-sectional survey in the United States in 2-year cycles. Both datasets collected information through face-to-face interviews, including demographic characteristics and laboratory tests.

**Figure 1.**
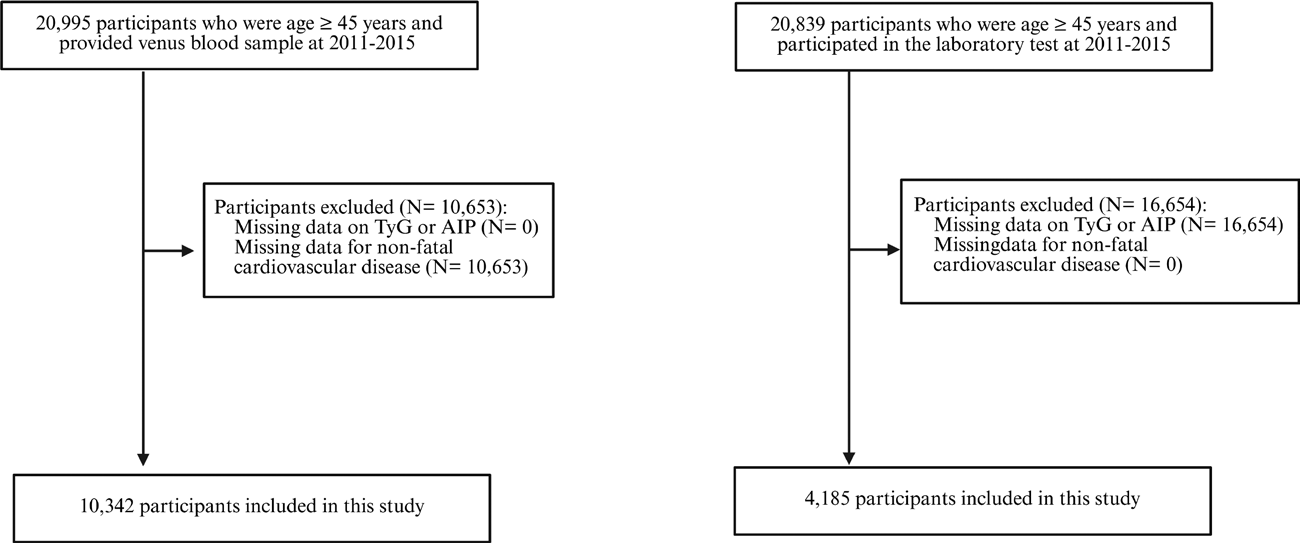
Flow chart for participant recruitment.

Participants aged ≥45 years were included if fasting blood sample data were available for the 2011–2016 survey period. Individuals with missing data on key variables (e.g., TyG index, AIP), CVD outcomes, or covariates were excluded. Finally, 10,342 participants from CHARLS and 4185 participants from NHANES were included in the final analysis (Fig. 1).

### 2.2. Ethics statement

The CHARLS protocol was approved by the Ethics Review Committee of Peking University (IRB No. 00001052-11015). NHANES was approved by the National Center for Health Statistics Ethics Review Board (Protocol No. 2011–17). All participants gave written informed consent.

### 2.3. Definitions of TyG, AIP, and TyG-AIP

The TyG index was calculated as ln [TG (mg/dL) × FBG (mg/dL)/ 2]. AIP was calculated as log10 [TG (mg/dL)/HDL-C (mg/dL)]. TyG-AIP was obtained by multiplying the TyG index by AIP. Participants were then divided into 4 groups (Q1–Q4) according to the quartiles of TyG index, AIP, and TyG-AIP. The lowest quartile was used as the reference.

### 2.4. Assessment of outcome

The main outcome was prevalent CVD, including heart disease and stroke. In CHARLS, participants were asked whether a doctor had ever told them that they had heart disease or stroke, as well as when the condition was first diagnosed. In NHANES, CVD was identified from the medical conditions questionnaire administered during the interview.

### 2.5. Assessment of covariates

Morning fasting blood samples were collected, and TG, HDL-C, and low-density lipoprotein cholesterol (LDL-C) were measured. Demographic and lifestyle information was obtained using standardized questionnaires. Age, sex, marital status, annual household income, and educational level were included as demographic variables. Smoking and alcohol consumption were included as lifestyle variables. Weight and height were measured for each participant. Body mass index (BMI) was calculated as weight (kg) divided by height squared (m^2^). Obesity was defined as a BMI of ≥28 kg/m^2^ in CHARLS, BMI of ≥30 kg/m^2^ in NHANES. Blood pressure was measured by physicians after training using the right arm of each participant in the seated position. Three measurements were taken, and their average was used. Hypertension was defined as using a blood pressure ≥140/90 mm Hg, use of antihypertensive medications, or a self-reported history of hypertension. Diabetes mellitus was identified through physical exams and defined as the use of hypoglycemic medications, a FBG concentration of ≥126 mg/dl (7.0 mmol/L), or glycosylated hemoglobin ≥6.5 % or self-reported diagnosis.

### 2.6. Statistical analysis

Normally distributed continuous variables are given as mean ± standard deviation. ANOVA was used to compare groups. Skewed continuous variables were expressed as the median (interquartile range) and compared using the Wilcoxon rank-sum test. Categorical variables were presented as counts (percentages), and comparisons were made using the chi-square test.

Multiple logistic regression models were used to assess odds ratios (ORs) and 95% confidence intervals (CIs) for the associations between the TyG index, AIP, TyG-AIP, and CVD. Model 1 was adjusted for age and sex. Model 2 was additionally adjusted for education level, married status, alcohol consumption, and smoking habits. Model 3 was further adjusted for systolic blood pressure (SBP), obesity status, and LDL-C. Restricted cubic spline (RCS) models were used to examine the relationship between TyG-related indices and CVD. Values below the 5th percentile and above the 95th percentile were excluded. Nonlinearity was tested with the likelihood ratio test. ROC analysis was performed for TyG index, AIP, and TyG-AIP, and the corresponding area under the curve (AUC) values were obtained.

Subgroup analyses were performed according to sex (male and female), and age (<60 vs ≥60 years, defined as elders by the World Health Organization^[27]^). Sensitivity analyses were done by excluding participants using lipid-lowering or antihypertensive medications, excluding those with diabetes, and additionally adjusting for ethnicity.

A two-sided *P* < .05 was considered to be statistically significant. All analyses were performed using the R program (version 4.4.1).

## 3. Results

### 3.1. Participants characteristics

Table 1 presents the characteristics of participants from the CHARLS and NHANES cohorts. There were 10,342 participants in CHARLS and 4185 in NHANES. The mean age was 59.43 ± 9.56 years in CHARLS and 62.18 ± 10.67 years in NHANES, and men accounted for 46.91% and 48.91% of the two cohorts, respectively. CVD was present in 1490 participants in CHARLS and 774 in NHANES. In both cohorts, participants with CVD were older and more likely to have higher TG, lower HDL-C, obesity, diabetes, and hypertension. A comparison between the included and excluded participants is presented in Supplementary Table S1.

**Table 1:** Characteristic of the study population.

Characteristics	CHARLS			NHANES		
	Total	No CVD	CVD	Total	No CVD	CVD
Participants, n	19,573	16,164	3409	4185	3441	774
Age (yr), mean ± SD	60.58 ± 9.45	59.99 ± 9.40	63.34 ± 9.19	62.18 ± 10.67	60.81 ± 10.35	68.52 ± 9.81
Sex, n (%)						
Women	10,461 (53.45)	8478 (52.45)	1983 (58.17)	2138 (51.09)	1805 (52.46)	333 (44.76)
Men	9112 (46.55)	7686 (47.55)	1426 (41.83)	2047 (48.91)	1636 (47.54)	411 (55.24)
Marital status, n (%)						
Live without a spouse	2573 (13.15)	2009 (12.43)	564 (16.54)	2560 (61.17)	2148 (62.42)	412 (55.38)
Live with spouse	17,000 (86.85)	14,155 (87.57)	2845 (83.46)	1625 (38.83)	1293 (37.58)	332 (44.62)
Education attainment, n (%)						
Middle school or below	13,459 (68.76)	11,080 (68.55)	2379 (69.79)	2505 (59.86)	2066 (60.04)	439 (59.01)
High school or above	6114 (31.24)	5084 (31.45)	1030 (30.21)	1680 (40.14)	1375 (39.96)	305 (40.99)
Tobacco smoking, n (%)						
Non-smoker	14,117 (72.12)	11,465 (70.93)	2652 (77.79)	3429 (81.94)	2847 (82.74)	582 (78.23)
Smoker	5456 (27.88)	4699 (29.07)	757 (22.21)	756 (18.06)	594 (17.26)	162 (21.77)
Alcohol consumption, n (%)						
Non-drinker	12,943 (66.13)	10,400 (64.34)	2543 (74.60)	1860 (44.44)	1460 (42.43)	400 (53.76)
Drinker	6630 (33.87)	5764 (35.66)	866 (25.40)	2325 (55.56)	1981 (57.57)	344 (46.24)
Obesity, n (%)						
No	17,154 (87.64)	14,376 (88.94)	2778 (81.49)	2549 (60.91)	2127 (61.81)	422 (56.72)
Yes	2419 (12.36)	1788 (11.06)	631 (18.51)	1636 (39.09)	1314 (38.19)	322 (43.28)
Hypertension, n (%)						
No	11,183 (57.13)	9904 (61.27)	1279 (37.52)	1663 (39.74)	1516 (44.06)	147 (19.76)
Yes	8390 (42.87)	6260 (38.73)	2130 (62.48)	2522 (60.26)	1925 (55.94)	597 (80.24)
Diabetes Mellitus, n (%)						
No	16,047 (81.99)	13,558 (83.88)	2489 (73.01)	2838 (67.81)	2451 (71.23)	387 (52.02)
Yes	3526 (18.01)	2606 (16.12)	920 (26.99)	1347 (32.19)	990 (28.77)	357 (47.98)
BMI (kg/m^2^), mean ± SD	23.73 ± 3.83	23.57 ± 3.71	24.53 ± 4.25	29.40 ± 6.67	29.23 ± 6.56	30.16 ± 7.13
SBP (mm Hg), mean ± SD	130.02 ± 20.87	129.20 ± 20.62	133.89 ± 21.58	128.90 ± 19.20	128.37 ± 18.65	131.34 ± 21.39
DBP (mm Hg), mean ± SD	75.86 ± 11.85	75.59 ± 11.77	77.16 ± 12.13	69.36 ± 12.06	70.10 ± 11.77	65.95 ± 12.79
LDL-C (mg/dL), mean ± SD	110.44 ± 33.05	110.65 ± 32.93	109.46 ± 33.60	114.45 ± 36.53	117.80 ± 35.59	98.96 ± 36.85
HDL-C (mg/dL), mean ± SD	51.30 ± 13.74	51.66 ± 13.77	49.62 ± 13.46	55.21 ± 17.16	56.02 ± 17.12	51.45 ± 16.84
TG (mg/dL), IQR	107.97 (77.88,158.41)	106.19 (76.99,154.88)	120.35 (85.84,173.45)	103.00 (74.00,151.00)	102.00 (73.00,150.00)	108.00 (78.00,158.00)
FBG (mg/dL), mean ± SD	105.67 ± 33.48	105.27 ± 33.01	107.58 ± 35.57	115.94 ± 40.01	114.17 ± 38.11	124.12 ± 46.98
AIP, IQR	−0.02 (−0.21,0.19)	−0.04 (−0.22,0.18)	0.04 (−0.15,0.25)	8.69 ± 0.66	8.67 ± 0.66	8.80 ± 0.70
TyG, mean ± SD	8.68 ± 0.64	8.65 ± 0.63	8.79 ± 0.66	−0.06 (−0.28,0.15)	−0.08 (−0.29,0.14)	0.00 (−0.23,0.20)
TyG-AIP, IQR	−0.19 (−1.75,1.70)	−0.31 (−1.84,1.59)	0.33 (−1.27,2.28)	−0.53 (−2.29,1.34)	−0.65 (−2.39,1.22)	−0.01 (−1.85,1.87)

AIP = atherogenic index of plasma, BMI = body mass index, CHARLS = China Health and Retirement Longitudinal Study, CVD = cardiovascular disease, DBP = diastolic blood pressure, FBG = fasting blood glucose, HDL-C = low-density lipoprotein cholesterol, IQR = interquartile range, LDL-C = low-density lipoprotein cholesterol, NHANES = National Health and Nutrition Examination Survey, SBP = systolic blood pressure, SD = standard deviation, TG = triglyceride, TyG-AIP = triglyceride-glucose index combined with the atherogenic index of plasma.

### 3.2. Associations between TyG index, AIP, TyG-AIP, and CVD

The logistic regression results are shown in Table 2 **and** Supplementary Tables S2S3. In both CHARLS and NHANES, participants in Q4 had higher CVD odds than those in Q1. For TyG-AIP, the ORs were 1.68 (95% CI: 1.43–1.98) in CHARLS and 1.97 (95% CI: 1.53–2.54) in NHANES. For the TyG index, the values were 1.66 (95% CI: 1.41–1.95) and 1.84 (95% CI: 1.43–2.37). For AIP, the values were 1.69 (95% CI: 1.43–1.99) and 1.97 (95% CI: 1.53–2.54).

**Table 2:** Cross-sectional association of TyG, AIP and TyG-AIP with risk of cardiovascular disease.

Characteristics	No. cases/ Total	OR (95% CI)		
		Model 1	Model 2	Model 3
CHARLS				
TyG				
Q1	681/ 4895	Ref	Ref	Ref
Q2	753/ 4902	1.10 (0.99,1.24)	1.09 (0.97,1.22)	1.08 (0.96,1.21)
Q3	919/ 4886	1.40 (1.26,1.56)	1.36 (1.22,1.52)	1.32 (1.18,1.47)
Q4	1056/ 4890	1.72 (1.54,1.91)	1.67 (1.50,1.86)	1.50 (1.34,1.67)
Per SD	/	1.24 (1.19,1.28)	1.23 (1.18,1.27)	1.17 (1.13,1.21)
AIP				
Q1	625/ 4893	Ref	Ref	Ref
Q2	790/ 4902	1.32 (1.18,1.48)	1.28 (1.14,1.43)	1.26 (1.12,1.41)
Q3	948/ 4884	1.66 (1.48,1.85)	1.59 (1.42,1.78)	1.52 (1.35,1.70)
Q4	1046/ 4894	1.96 (1.75,2.19)	1.87 (1.67,2.08)	1.65 (1.48,1.85)
Per SD	/	1.28 (1.23,1.32)	1.26 (1.21,1.31)	1.19 (1.15,1.24)
TyG-AIP				
Q1	627/ 4893	Ref	Ref	Ref
Q2	785/ 4892	1.31 (1.17,1.47)	1.27 (1.13,1.42)	1.25 (1.11,1.40)
Q3	948/ 4895	1.65 (1.47,1.84)	1.58 (1.41,1.77)	1.51 (1.35,1.69)
Q4	1049/ 4893	1.96 (1.75,2.18)	1.86 (1.67,2.08)	1.65 (1.47,1.85)
Per SD	/	1.03 (1.03,1.03)	1.03 (1.02,1.03)	1.02 (1.02,1.03)
NHANES				
TyG				
Q1	149/ 1043	Ref	Ref	Ref
Q2	176/ 1053	1.12 (0.87,1.43)	1.14 (0.89,1.46)	1.27 (0.99,1.65)
Q3	184/ 1041	1.28 (1.00,1.63)	1.26 (0.98,1.61)	1.38 (1.07,1.78)
Q4	235/ 1048	1.77 (1.40,2.24)	1.76 (1.39,2.24)	1.84 (1.43,2.37)
Per SD	/	1.25 (1.15,1.35)	1.24 (1.14,1.35)	1.24 (1.14,1.36)
AIP				
Q1	150/ 1043	Ref	Ref	Ref
Q2	160/ 1053	1.1 (0.86,1.42)	1.11 (0.86,1.43)	1.21 (0.93,1.56)
Q3	204/ 1046	1.48 (1.17,1.89)	1.45 (1.14,1.85)	1.54 (1.19,1.98)
Q4	230/ 1047	1.97 (1.55,2.50)	1.89 (1.49,2.41)	1.97 (1.53,2.54)
Per SD	/	1.30 (1.19,1.41)	1.28 (1.17,1.39)	1.28 (1.17,1.40)
TyG-AIP				
Q1	150/ 1047	Ref	Ref	Ref
Q2	160/ 1045	1.10 (0.86,1.42)	1.11 (0.86,1.43)	1.20 (0.93,1.55)
Q3	204/ 1047	1.48 (1.17,1.89)	1.45 (1.13,1.85)	1.53 (1.19,1.97)
Q4	230/ 1046	1.97 (1.55,2.51)	1.90 (1.49,2.42)	1.97 (1.53,2.54)
Per SD	/	1.30 (1.19,1.41)	1.28 (1.17,1.39)	1.27 (1.17,1.39)

Model 1: adjusted for age, sex.

Model 2 further adjusted for education level, married status, smoking, and drinking habits.

Model 3 further adjusted for SBP, obesity, and LDL-C.

AIP = atherogenic index of plasma, CHARLS = China Health and Retirement Longitudinal Study, CI = confidence interval, NHANES = National Health and Nutrition Examination Survey, OR = odds ratio, SD = standard deviation, TyG = triglyceride-glucose, TyG-AIP = triglyceride-glucose index combined with the atherogenic index of plasma.

### 3.3. Restricted cubic spline analysis (RCS) of TyG-related indices and CVD

**Figure 2.**
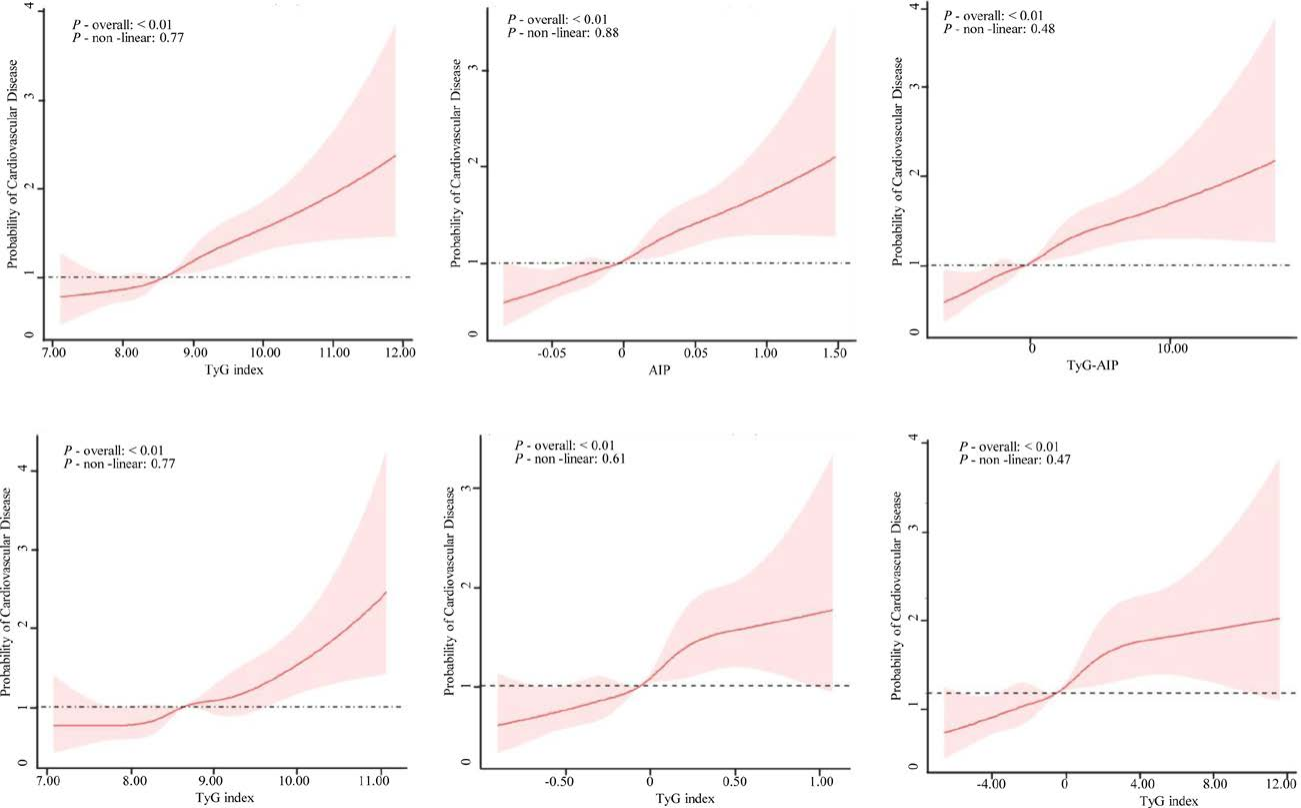
Association between TyG, AIP, and TyG-AIP with cardiovascular disease was evaluated by RCS (Top: CHARLS; Bottom: NHANES). Model adjusted for the age, sex, education, married status, smoking habits, drinking habits, SBP, LDL-C, and obesity. AIP = atherogenic index of plasma, CHARLS = China Health and Retirement Longitudinal Study, LDL-C = low-density lipoprotein cholesterol, NHANES = National Health and Nutrition Examination Survey, RCS = restricted cubic spline, SBP = systolic blood pressure, TyG = triglyceride-glucose.

Figure 2 shows the restricted cubic spline analyses evaluating the associations between TyG-related indices and CVD. After adjustment for covariates in Model 3, linear associations were observed between TyG index, AIP, TyG-AIP, and the odds of CVD (*P* for overall < .01; *P* for nonlinearity > .05).

### 3.4. ROC curve analysis of TyG-related indices for CVD

**Figure 3.**
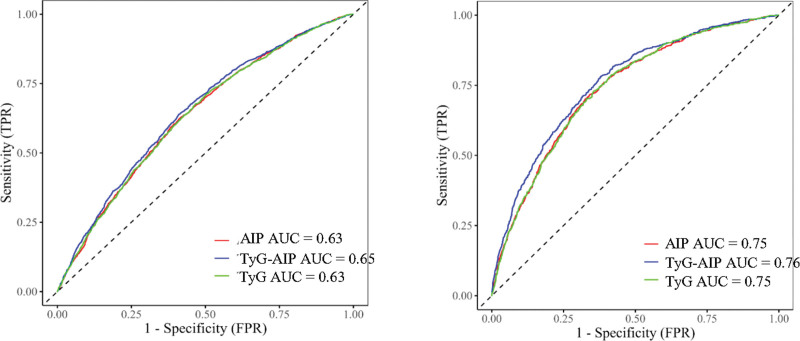
Receiver operating characteristic (ROC) curves of TyG, AIP, TyG-AIP in relation to cardiovascular disease (Left: CHARLS, Right: NHANES). Model adjust for age, sex, education level, married status, smoking habits, drinking habits, SBP, LDL-C, and obesity. AIP = atherogenic index of plasma, CHARLS = China Health and Retirement Longitudinal Study, LDL-C = low-density lipoprotein cholesterol, NHANES = National Health and Nutrition Examination Survey, ROC = receiver operating characteristic, SBP = systolic blood pressure, TyG = triglyceride-glucose.

The ROC results are shown in Figure 3. In both CHARLS and NHANES, TyG-AIP had the largest AUC, at 0.65 (95% CI: 0.63–0.66) and 0.76 (95% CI: 0.74–0.78), respectively. The AUCs for the TyG index were 0.64 (95% CI: 0.62–0.65) in CHARLS and 0.75 (95% CI: 0.74–0.76) in NHANES, while those for AIP were 0.64 (95% CI: 0.62–0.65) and 0.75 (95% CI: 0.72–0.76).

### 3.5. Stratified and sensitivity analyses

The results of subgroup analyses are presented in Supplementary Tables S4s6s7s8s9S9. Across most subgroups, the Q4 group showed higher CVD odds than the Q1 group for TyG index, AIP, and TyG-AIP. The results changed little after excluding participants using glucose-lowering or lipid-lowering medications, excluding those with diabetes, or adding ethnicity to the model (Supplementary Table S10).

## 4. Discussion

Our findings showed that higher TyG index, AIP, and TyG-AIP levels were positively associated with CVD in middle-aged and older adults from China and the United States. Among these 3 indicators, TyG-AIP showed the best ability to distinguish individuals with CVD.

A positive association between higher TyG index levels and CVD was found in middle-aged and older adults from both Chinese and U.S. populations. The highest TyG quartile had a higher CVD risk than the lowest quartile, which agrees with earlier findings.^[28,29]^ A large longitudinal cohort study from South Korea reported that individuals in the Highest TyG index quartile had an increased risk of CVD in the general population (HR, 1.282, 95%CI 1.261-1.303).^[29]^ Similarly, we also found that AIP was positively associated with CVD, which was consistent with a previous study in patients with type 2 diabetes mellitus.^[30]^ The RCS analysis showed linear relationships of the TyG index and AIP with CVD risk. Similar linear associations have also been reported in general populations for both the TyG index and AIP.^[18,20]^ However, previous studies were primarily conducted in general populations. Middle-aged and older adults often have more cardiometabolic risk factors. This may make the association between metabolic indices and CVD stronger. Therefore, our results further support previous evidence by showing consistent linear associations of TyG and AIP with CVD across nationally representative populations from China and the United States.

To our knowledge, this study is among the first to examine the association between the combined TyG-AIP index and CVD. We observed that higher TyG-AIP levels were associated with higher odds of CVD. Although limited studies have investigated the joint effects of TyG and AIP on CVD outcomes, previous research has evaluated combinations of TyG with obesity-related indicators, such as BMI and waist circumference.^[21,31,32]^ Ren *et al* reported that individuals with persistently elevated TyG-WHtR had a 58% higher risk of CVD compared with those with low and stable TyG-WHtR (OR 1.58, 95% CI: 1.16–2.15) in a middle-aged and older Chinese population.^[22]^ In the present study, TyG-AIP showed better discriminatory ability for CVD than either TyG or AIP alone. Similar improvements in predictive performance have been observed when combining TyG with anthropometric indices. A study involving 1145 participants from Korea also showed that the triglyceride-glucose index combined with waist circumference had greater diagnostic accuracy for coronary artery calcification progression than TyG alone.^[32]^ Collectively, these findings suggest that integrating multiple metabolic indicators may provide a more comprehensive assessment of cardiometabolic risk.

TyG index and AIP may be related to higher CVD risk because they are linked to metabolic problems such as dyslipidemia, hypertension, and metabolic syndrome.^[33,34]^ These indices are simple markers of insulin resistance, and using them together may show cardiometabolic dysfunction better than using either one alone. These findings may be related to abnormal glucose and lipid metabolism. This may further damage blood vessels and promote atherosclerosis. Our results suggest that TyG-related indices may have value in CVD risk assessment.

It is still unclear why TyG-related indices are associated with CVD, but several mechanisms may be involved. Insulin resistance may be one reason why cardiometabolic disorders and CVD often occur together. Genes such as *RAGE, APM1,* and *ADIPOQ,* which are related to glucose and lipid metabolism, may play a role in both insulin resistance and CVD.^[35–37]^ Mendelian randomization studies suggest that insulin resistance may have a causal link with CVD.^[38]^ TyG index and AIP are both related to insulin resistance. Therefore, high values of these indices may point to abnormal lipid and glucose metabolism, which may increase CVD risk.^[39,40]^ Insulin resistance may also be linked to chronic low-grade inflammation. Tumor necrosis factor-α, interleukin-6, and interleukin-1 may all increase.^[41,42]^ This may further damage the vascular endothelium and increase CVD risk.^[43]^

Our study used data from China and the United States, which allowed us to examine the association in 2 different populations. Still, several limitations need to be considered. First, this study included only middle-aged and older adults, so the findings may not apply to younger people. Second, CVD was identified from self-reported physician diagnosis, which may have led to underreporting or misclassification. Third, TG, HDL-C, and FBG were measured only once, so they may not fully represent long-term metabolic status. Finally, because this was a cross-sectional analysis, we could not determine causality. In the future, more prospective studies are needed to examine how long-term exposure to TyG-related indices is related to CVD and what biological mechanisms may be involved.

## 5. Conclusion

The study demonstrated linear associations between higher TyG index, AIP, and TyG-AIP levels and increased CVD risk in middle-aged and older populations. Of the indicators evaluated, TyG-AIP showed the greatest ability to distinguish individuals with CVD.

## Acknowledgment

The authors thank the CHARLS and NHANES study teams for their valuable contributions and all participants for providing the data used in this study.

The authors also thank Dr Jing Zhang (Second Department of Infectious Diseases, Shanghai Fifth People’s Hospital, Fudan University) for his important work on the CHARLS and NHANES databases. His development of the *nhanesR* and *charlsR* packages and related web resources greatly facilitated data access and analysis in the present study.

## Author contributions

**Investigation:** Yuanfeng Wu, Yingni Chen, Xianxuan Wang.

**Methodology:** Yuanfeng Wu, Yingni Chen, Xianxuan Wang.

**Resources:** Yuanfeng Wu, Qiang Wu, Xianxuan Wang.

**Visualization:** Yuanfeng Wu.

**Project administration:** Yingni Chen, Xianxuan Wang.

**Software:** Qiang Wu, Chulin Wang, Xianxuan Wang.

**Validation:** Chulin Wang, Xianxuan Wang.

**Data curation:** Xianxuan Wang.

**Formal analysis:** Xianxuan Wang.

**Supervision:** Xianxuan Wang.

**Writing – original draft:** Yuanfeng Wu, Xianxuan Wang.

**Writing – review & editing:** Yuanfeng Wu, Xianxuan Wang.




















