# Supplementary material for: Association between triglyceride-glucose-atherogenic index of plasma and cardiovascular disease in middle-aged and older Chinese and American individuals: A cross-sectional analysis of two nationwide cohort datasets
Source: Medicine (Baltimore). 2026 May 8;105(19):e48675. doi: 10.1097/MD.0000000000048675 (PMC13166467; doi:10.1097/MD.0000000000048675)
Supplement: Supplementary file 1 [file medi-105-e48675-s001.docx]

**Table S1** Baseline characteristics of the participants excluded and included in analyses

| Characteristics | CHARLS | | |  | NHANES | | |
| --- | --- | --- | --- | --- | --- | --- | --- |
|  | Total | Excluded | Included |  | Total | Excluded | Included |
| Participants, n | 20,995 | 10,653 | 10,342 |  | 20839 | 16654 | 4185 |
| Age (year), mean ± SD | 60.10 ± 9.63 | 60.75 ± 9.65 | 59.43 ± 9.56 |  | 62.50 ± 10.97 | 62.58 ± 11.05 | 62.18 ± 10.67 |
| Sex, n (%) |  |  |  |  |  |  |  |
| Women | 11171(53.21) | 5680(53.32) | 5491(53.09) |  | 10551(50.63) | 8413(50.52) | 2138(51.09) |
| Men | 9824(46.79) | 4973(46.68) | 4851(46.91) |  | 10288(49.37) | 8241(49.48) | 2047(48.91) |
| Marital status, n (%) |  |  |  |  |  |  |  |
| Live without spouse | 2706(12.89) | 1442(13.54) | 1264(12.22) |  | 12628(60.64) | 10068(60.51) | 2560(61.17) |
| Live with spouse | 18289(87.11) | 9211(86.46) | 9078(87.78) |  | 8196(39.36) | 6571(39.49) | 1625(38.83) |
| Education attainment, n (%) |  |  |  |  |  |  |  |
| Middle school or below | 13835(68.58) | 6719(68.34) | 7116(68.81) |  | 16763(80.56) | 14258(85.77) | 2505(59.86) |
| High school or above | 6339(31.42) | 3113(31.66) | 3226(31.19) |  | 4046(19.44) | 2366(14.23) | 1680(40.14) |
| Tobacco smoking, n (%) |  |  |  |  |  |  |  |
| Non-smoker | 15088(72.02) | 7706(72.64) | 7382(71.38) |  | 17166(82.42) | 13737(82.54) | 3429(81.94) |
| Smoker | 5863(27.98) | 2903(27.36) | 2960(28.62) |  | 3662(17.58) | 2906(17.46) | 756(18.06) |
| Alcohol consumption, n (%) |  |  |  |  |  |  |  |
| Non-drinker | 13755(65.65) | 6861(64.67) | 6894(66.66) |  | 7293(38.78) | 5433(37.16) | 1860(44.44) |
| Drinker | 7196(34.35) | 3748(35.33) | 3448(33.34) |  | 11514(61.22) | 9189(62.84) | 2325(55.56) |
| Obesity^a^, n (%) |  |  |  |  |  |  |  |
| No | 18168(87.59) | 9014(86.68) | 9154(88.51) |  | 12379(60.29) | 9830(60.13) | 2549(60.91) |
| Yes | 2573(12.41) | 1385(13.32) | 1188(11.49) |  | 8155(39.71) | 6519(39.87) | 1636(39.09) |
| Hypertension, n (%) |  |  |  |  |  |  |  |
| No | 13602(65.55) | 7347(70.58) | 6255(60.48) |  | 8550(41.05) | 6887(41.38) | 1663(39.74) |
| Yes | 7150(34.45) | 3063(29.42) | 4087(39.52) |  | 12280(58.95) | 9758(58.62) | 2522(60.26) |
| Diabetes Mellitus, n (%) |  |  |  |  |  |  |  |
| No | 17645(84.04) | 9005(84.53) | 8640(83.54) |  | 14881(71.41) | 12043(72.31) | 2838(67.81) |
| Yes | 3350(15.96) | 1648(15.47) | 1702(16.46) |  | 5958(28.59) | 4611(27.69) | 1347(32.19) |
| BMI (kg/m2), mean ± SD | 23.74 ± 3.81 | 23.95 ± 3.71 | 23.53 ± 3.90 |  | 29.46 ± 6.64 | 29.48 ± 6.62 | 29.39 ± 6.69 |
| SBP (mmHg), mean ± SD | 129.81 ± 20.81 | 129.01 ± 20.03 | 130.62 ± 21.54 |  | 129.75 ± 19.78 | 130.00 ± 19.94 | 128.80 ± 19.13 |
| DBP (mmHg), mean ± SD | 75.89 ± 11.84 | 76.03 ± 11.54 | 75.75 ± 12.13 |  | 70.62 ± 12.45 | 70.99 ± 12.52 | 69.23 ± 12.06 |
| HDL-C | 109.83 ± 32.83 | 102.82 ± 29.14 | 117.04 ± 34.79 |  | 115.36 ± 36.95 | 116.07 ± 37.17 | 114.38 ± 36.63 |
| LDL-C (mg/dL), mean ± SD | 51.26 ± 13.57 | 51.39 ± 11.66 | 51.14 ± 15.29 |  | 53.92 ± 16.68 | 53.59 ± 16.55 | 55.21 ± 17.16 |
| TG (mg/dL), IQR | 107.97(77.88,159.29) | 111.50(81.42,163.72) | 105.32(75.22,153.99) |  | 109.00(78.00,157.00) | 113.00(80.00,162.00) | 103.00(74.00,151.00) |
| FBG (mg/dL), mean ± SD | 105.25 ± 33.17 | 100.75 ± 30.43 | 109.89 ± 35.18 |  | 116.43 ± 40.44 | 116.76 ± 40.74 | 115.94 ± 40.01 |
| AIP, IQR | -0.02(-0.21,0.19) | -0.01(-0.18,0.19) | -0.03(-0.24,0.19) |  | -0.06(-0.28,0.15) | 0.04(-0.22,0.15) | -0.06(-0.28,0.15) |
| TyG, mean ± SD | 8.68 ± 0.64 | 8.67 ± 0.62 | 8.68 ± 0.66 |  | 2.54 ± 3.10 | 1.00 ± 0.00 | 8.69 ± 0.66 |
| TyG-AIP, IQR | -0.18(-1.73,1.72) | -0.08(-1.52,1.71) | -0.30(-1.97,1.73) |  | -0.53(-2.29,1.34) | 0.04(-0.22,0.15) | -0.53(-2.29,1.34) |

Abbreviation: AIP, atherogenic index of plasma; BMI, body mass index; CMD, cardiometabolic disease; DBP, diastolic blood pressure; HDL-C: low-density lipoprotein cholesterol; hs-CRP, hypersensitivity C reactive protein; SBP, systolic blood pressure; TG, triglyceride.
