# Supplementary material for: Association between triglyceride-glucose-atherogenic index of plasma and cardiovascular disease in middle-aged and older Chinese and American individuals: A cross-sectional analysis of two nationwide cohort datasets
Source: Medicine (Baltimore). 2026 May 8;105(19):e48675. doi: 10.1097/MD.0000000000048675 (PMC13166467; doi:10.1097/MD.0000000000048675)
Supplement: Supplementary file 2 [file medi-105-e48675-s002.docx]

**Table S2.** Cross-sectional association of TyG, AIP and TyG-AIP with risk of CVD subgroups in **CHARLS**

| Characteristics | No. cases / Total | OR (95% CI) | | |
| --- | --- | --- | --- | --- |
|  |  | Model 1 | Model 2 | Model 3 |
| **Heart disease** |  |  |  |  |
| **TyG** |  |  |  |  |
| Q1 | 252 / 2579 | 1.00 (Reference) | 1.00 (Reference) | 1.00 (Reference) |
| Q2 | 290 / 2595 | 1.14 (0.95, 1.37) | 1.12 (0.94, 1.34) | 1.10 (0.92, 1.32) |
| Q3 | 324 / 2582 | 1.28 (1.07, 1.52) | 1.24 (1.04, 1.48) | 1.19 (1.00, 1.43) |
| Q4 | 438 / 2586 | 1.86 (1.57, 2.19) | 1.81 (1.53, 2.14) | 1.68 (1.41, 1.99) |
| Per SD | / | 1.27 (1.20, 1.34) | 1.26 (1.19, 1.33) | 1.23 (1.16, 1.30) |
| **AIP** |  |  |  |  |
| Q1 | 248 / 2588 | 1.00 (Reference) | 1.00 (Reference) | 1.00 (Reference) |
| Q2 | 298 / 2579 | 1.23 (1.03, 1.47) | 1.19 (0.99, 1.42) | 1.17 (0.97, 1.40) |
| Q3 | 337 / 2587 | 1.42 (1.19, 1.69) | 1.36 (1.14, 1.62) | 1.29 (1.08, 1.54) |
| Q4 | 421 / 2588 | 1.90 (1.60, 2.24) | 1.81 (1.53, 2.15) | 1.68 (1.41, 1.99) |
| Per SD | / | 1.27 (1.20, 1.35) | 1.26 (1.19, 1.33) | 1.22 (1.15, 1.30) |
| **TyG-AIP** |  |  |  |  |
| Q1 | 249 / 2586 | 1.00 (Reference) | 1.00 (Reference) | 1.00 (Reference) |
| Q2 | 295 / 2585 | 1.21 (1.01, 1.44) | 1.17 (0.97, 1.40) | 1.14 (0.95, 1.37) |
| Q3 | 340 / 2586 | 1.43 (1.20, 1.70) | 1.37 (1.15, 1.63) | 1.30 (1.08, 1.55) |
| Q4 | 420 / 2585 | 1.88 (1.59, 2.23) | 1.80 (1.52, 2.13) | 1.67 (1.40, 1.98) |
| Per SD | / | 1.02 (1.02, 1.03) | 1.02 (1.02, 1.03) | 1.02 (1.01, 1.03) |
| **Stroke** |  |  |  |  |
| **TyG** |  |  |  |  |
| Q1 | 49 / 2579 | 1.00 (Reference) | 1.00 (Reference) | 1.00 (Reference) |
| Q2 | 55 / 2595 | 1.16 (0.78, 1.71) | 1.14 (0.77, 1.69) | 1.14 (0.77, 1.68) |
| Q3 | 56 / 2582 | 1.18 (0.80, 1.75) | 1.16 (0.79, 1.72) | 1.13 (0.76, 1.69) |
| Q4 | 83 / 2586 | 1.84 (1.28, 2.65) | 1.80 (1.25, 2.59) | 1.67 (1.16, 2.42) |
| Per SD |  | 1.23 (1.09, 1.38) | 1.22 (1.08, 1.37) | 1.18 (1.04, 1.33) |
| **AIP** |  |  |  |  |
| Q1 | 39 / 2588 | 1.00 (Reference) | 1.00 (Reference) | 1.00 (Reference) |
| Q2 | 53 / 2579 | 1.43 (0.94, 2.17) | 1.41 (0.93, 2.15) | 1.41 (0.93, 2.15) |
| Q3 | 76 / 2587 | 2.11 (1.42, 3.12) | 2.06 (1.39, 3.05) | 1.97 (1.32, 2.94) |
| Q4 | 75 / 2588 | 2.18 (1.47, 3.24) | 2.12 (1.42, 3.14) | 2.00 (1.35, 2.99) |
| Per SD |  | 1.33 (1.18, 1.49) | 1.31 (1.16, 1.48) | 1.27 (1.12, 1.44) |
| **TyG-AIP** |  |  |  |  |
| Q1 | 39 / 2586 | 1.00 (Reference) | 1.00 (Reference) | 1.00 (Reference) |
| Q2 | 53 / 2585 | 1.42 (0.94, 2.16) | 1.40 (0.92, 2.14) | 1.40 (0.92, 2.14) |
| Q3 | 76 / 2586 | 2.11 (1.42, 3.12) | 2.06 (1.39, 3.05) | 1.96 (1.32, 2.93) |
| Q4 | 75 / 2585 | 2.18 (1.47, 3.23) | 2.11 (1.42, 3.14) | 2.00 (1.34, 2.98) |
| Per SD |  | 1.03 (1.02, 1.04) | 1.03 (1.01, 1.04) | 1.02 (1.01, 1.04) |

Model 1: adjusted for age, sex,

Model 2 further adjusted for education level, married status, smoking and drinking habits.

Model 3 further adjusted for SBP, DBP, obesity, TC.
