# Supplementary material for: Association between triglyceride-glucose-atherogenic index of plasma and cardiovascular disease in middle-aged and older Chinese and American individuals: A cross-sectional analysis of two nationwide cohort datasets
Source: Medicine (Baltimore). 2026 May 8;105(19):e48675. doi: 10.1097/MD.0000000000048675 (PMC13166467; doi:10.1097/MD.0000000000048675)
Supplement: Supplementary file 3 [file medi-105-e48675-s003.docx]

**Table S4.** Stratified analysis for association of TyG with CVD in **CHARLS**

|  | OR (95%CI) | | | |  |
| --- | --- | --- | --- | --- | --- |
|  | Q1 | Q2 | Q3 | Q4 | *P*-interaction |
| Sex |  |  |  |  | 0.45 |
| Male | 1.00 (Reference) | 1.11 (0.88, 1.40) | 1.37 (1.09, 1.71) | 1.91 (1.54, 2.37) |  |
| Female | 1.00 (Reference) | 1.19 (0.94, 1.52) | 1.20 (0.94, 1.54) | 1.64 (1.30, 2.08) |  |
| Marital status |  |  |  |  | 0.61 |
| Live without spouse | 1.00 (Reference) | 1.11 (0.92, 1.33) | 1.29 (1.08, 1.54) | 1.81 (1.53, 2.14) |  |
| Live with spouse | 1.00 (Reference) | 1.52 (0.99, 2.34) | 1.59 (1.05, 2.42) | 2.13 (1.41, 3.25) |  |
| Education attainment |  |  |  |  | 0.61 |
| Middle school or below | 1.00 (Reference) | 1.11 (0.91, 1.35) | 1.25 (1.03, 1.52) | 1.76 (1.46, 2.12) |  |
| High school or above | 1.00 (Reference) | 1.34 (0.97, 1.86) | 1.59 (1.16, 2.18) | 2.13 (1.59, 2.89) |  |
| Tobacco smoking |  |  |  |  | 0.86 |
| Non-smoker | 1.00 (Reference) | 1.10 (0.91, 1.34) | 1.30 (1.07, 1.57) | 1.78 (1.48, 2.13) |  |
| Smoker | 1.00 (Reference) | 1.29 (0.93, 1.78) | 1.33 (0.96, 1.85) | 1.87 (1.37, 2.56) |  |
| Alcohol consumption |  |  |  |  | 0.58 |
| Non-drinker | 1.00 (Reference) | 1.22 (1.00, 1.49) | 1.34 (1.10, 1.63) | 1.88 (1.56, 2.27) |  |
| Drinker | 1.00 (Reference) | 0.93 (0.68, 1.28) | 1.15 (0.84, 1.58) | 1.61 (1.21, 2.16) |  |
| Obesity |  |  |  |  | 0.63 |
| No | 1.00 (Reference) | 1.17 (0.98, 1.40) | 1.27 (1.07, 1.51) | 1.73 (1.46, 2.05) |  |
| Yes | 1.00 (Reference) | 0.88 (0.50, 1.56) | 1.27 (0.76, 2.19) | 1.56 (0.97, 2.61) |  |

Model adjusted for age, sex, education level, married status, smoking and drinking habits, SBP, obesity, LDL-C.
