# Supplementary material for: Association between triglyceride-glucose-atherogenic index of plasma and cardiovascular disease in middle-aged and older Chinese and American individuals: A cross-sectional analysis of two nationwide cohort datasets
Source: Medicine (Baltimore). 2026 May 8;105(19):e48675. doi: 10.1097/MD.0000000000048675 (PMC13166467; doi:10.1097/MD.0000000000048675)
Supplement: Supplementary file 4 [file medi-105-e48675-s004.docx]

**Table S10.** Sensitivity analysis Cross-sectional association of TyG, RC and TyG-RC with risk of CVD

|  | Sensitivity 1 | Sensitivity 2 | Sensitivity 3 |
| --- | --- | --- | --- |
| **CHARLS** |  |  |  |
| **TyG** |  |  |  |
| Q1 | 1.00 (Reference) | 1.00 (Reference) | 1.00 (Reference) |
| Q2 | 1.17 (0.98, 1.41) | 1.11 (0.94, 1.32) | 1.16 (0.96, 1.40) |
| Q3 | 1.14 (0.94, 1.37) | 1.21 (1.02, 1.43) | 1.22 (1.01, 1.48) |
| Q4 | 1.42 (1.18, 1.71) | 1.65 (1.41, 1.95) | 1.56 (1.30, 1.88) |
| Per SD | 1.12 (1.04, 1.19) | 1.21 (1.15, 1.28) | 1.18 (1.11, 1.26) |
| **AIP** |  |  |  |
| Q1 | 1.00 (Reference) | 1.00 (Reference) | 1.00 (Reference) |
| Q2 | 1.16 (0.96, 1.40) | 1.19 (1.01, 1.42) | 1.18 (0.97, 1.42) |
| Q3 | 1.34 (1.11, 1.61) | 1.36 (1.15, 1.61) | 1.32 (1.09, 1.60) |
| Q4 | 1.40 (1.16, 1.69) | 1.69 (1.43, 1.99) | 1.61 (1.34, 1.94) |
| Per SD | 1.14 (1.06, 1.21) | 1.22 (1.15, 1.29) | 1.19 (1.12, 1.27) |
| **TyG-AIP** |  |  |  |
| Q1 | 1.00 (Reference) | 1.00 (Reference) | 1.00 (Reference) |
| Q2 | 1.13 (0.94, 1.36) | 1.17 (0.99, 1.39) | 1.15 (0.95, 1.39) |
| Q3 | 1.33 (1.11, 1.60) | 1.37 (1.16, 1.62) | 1.30 (1.08, 1.57) |
| Q4 | 1.39 (1.15, 1.68) | 1.68 (1.43, 1.98) | 1.60 (1.33, 1.93) |
| Per SD | 1.01 (1.01, 1.02) | 1.02 (1.01, 1.03) | 1.02 (1.01, 1.03) |
| **NHANES** |  |  |  |
| **TyG** |  |  |  |
| Q1 | 1.00 (Reference) | 1.00 (Reference) | 1.00 (Reference) |
| Q2 | 1.50 (0.97, 2.32) | 1.31 (1.01, 1.70) | 1.14 (0.87, 1.50) |
| Q3 | 1.69 (1.08, 2.62) | 1.45 (1.12, 1.88) | 1.30 (0.99, 1.70) |
| Q4 | 2.05 (1.30, 3.25) | 1.95 (1.50, 2.52) | 1.73 (1.33, 2.26) |
| Per SD | 1.24 (1.06, 1.46) | 1.27 (1.16, 1.39) | 1.24 (1.13, 1.36) |
| **AIP** |  |  |  |
| Q1 | 1.00 (Reference) | 1.00 (Reference) | 1.00 (Reference) |
| Q2 | 1.00 (0.64, 1.56) | 1.26 (0.97, 1.63) | 1.18 (0.89, 1.55) |
| Q3 | 1.48 (0.96, 2.28) | 1.64 (1.27, 2.12) | 1.40 (1.07, 1.84) |
| Q4 | 1.75 (1.13, 2.70) | 2.09 (1.61, 2.72) | 1.99 (1.51, 2.61) |
| Per SD | 1.27 (1.09, 1.48) | 1.3 (1.19, 1.43) | 1.29 (1.17, 1.42) |
| **TyG-AIP** |  |  |  |
| Q1 | 1.00 (Reference) | 1.00 (Reference) | 1.00 (Reference) |
| Q2 | 1.05 (0.67, 1.63) | 1.25 (0.96, 1.62) | 1.16 (0.88, 1.53) |
| Q3 | 1.50 (0.97, 2.32) | 1.63 (1.26, 2.11) | 1.40 (1.07, 1.84) |
| Q4 | 1.80 (1.16, 2.79) | 2.09 (1.61, 2.72) | 1.93 (1.47, 2.54) |
| Per SD | 1.25 (1.08, 1.45) | 1.30 (1.18, 1.42) | 1.04 (1.02, 1.05) |

Analysis 1: Excluding participants with hypertension (n = 9273 in **CHARLS**, n = 2453 in **NHANES**)

Analysis 2: Model adjusted for age, sex, education level, married status, smoking, drinking, SBP, obesity, LDL-C, and **ethnicity**.

Analysis 3: Excluding participants with missing co-variables (n = 8601 in **CHARLS**, n = 3671 in **NHANES.**)

Model adjusted for age, sex, education level, married status, smoking, drinking, SBP, obesity, and LDL-C, excepted for analysis 2.
