# Supplementary material for: Association between triglyceride-glucose-atherogenic index of plasma and cardiovascular disease in middle-aged and older Chinese and American individuals: A cross-sectional analysis of two nationwide cohort datasets
Source: Medicine (Baltimore). 2026 May 8;105(19):e48675. doi: 10.1097/MD.0000000000048675 (PMC13166467; doi:10.1097/MD.0000000000048675)
Supplement: Supplementary file 5 [file medi-105-e48675-s005.docx]

**Table S3.** Cross-sectional association of TyG, AIP and TyG-AIP with risk of CVD subgroups in **NHANES**

| Characteristics | No. cases / Total | OR (95% CI) | | |
| --- | --- | --- | --- | --- |
|  |  | Model 1 | Model 2 | Model 3 |
| **Heart disease** |  |  |  |  |
| **TyG** |  |  |  |  |
| Q1 | 110 / 1043 | 1.00 (Reference) | 1.00 (Reference) | 1.00 (Reference) |
| Q2 | 133 / 1053 | 1.14 (0.86, 1.50) | 1.16 (0.88, 1.53) | 1.29 (0.97, 1.72) |
| Q3 | 145 / 1041 | 1.37 (1.04, 1.80) | 1.36 (1.03, 1.79) | 1.48 (1.12, 1.97) |
| Q4 | 192 / 1048 | 1.94 (1.50, 2.53) | 1.94 (1.49, 2.53) | 2.00 (1.51, 2.63) |
| Per SD | / | 1.29 (1.18, 1.42) | 1.29 (1.18, 1.41) | 1.28 (1.18, 1.40) |
| **AIP** |  |  |  |  |
| Q1 | 104 / 1048 | 1.00 (Reference) | 1.00 (Reference) | 1.00 (Reference) |
| Q2 | 126 / 1044 | 1.27 (0.96, 1.69) | 1.29 (0.97, 1.72) | 1.40 (1.05, 1.88) |
| Q3 | 171 / 1046 | 1.83 (1.39, 2.39) | 1.8 (1.37, 2.36) | 1.90 (1.43, 2.51) |
| Q4 | 179 / 1047 | 2.17 (1.65, 2.84) | 2.11 (1.60, 2.77) | 2.15 (1.62, 2.86) |
| Per SD | / | 1.35 (1.23, 1.49) | 1.34 (1.22, 1.47) | 1.32 (1.22, 1.46) |
| **TyG-AIP** |  |  |  |  |
| Q1 | 104 / 1047 | 1.00 (Reference) | 1.00 (Reference) | 1.00 (Reference) |
| Q2 | 126 / 1045 | 1.27 (0.96, 1.69) | 1.29 (0.97, 1.71) | 1.39 (1.04, 1.86) |
| Q3 | 171 / 1047 | 1.83 (1.39, 2.39) | 1.80 (1.37, 2.36) | 1.89 (1.43, 2.51) |
| Q4 | 179 / 1046 | 2.17 (1.66, 2.85) | 2.11 (1.61, 2.77) | 2.15 (1.62, 2.86) |
| Per SD | / | 1.34 (1.23, 1.47) | 1.32 (1.21, 1.45) | 1.31 (1.19, 1.44) |
| **Stroke** |  |  |  |  |
| **TyG** |  |  |  |  |
| Q1 | 67 / 1043 | 1.00 (Reference) | 1.00 (Reference) | 1.00 (Reference) |
| Q2 | 69 / 1053 | 0.96 (0.67, 1.36) | 0.97 (0.68, 1.39) | 1.05 (0.73, 1.50) |
| Q3 | 62 / 1041 | 0.9 (0.62, 1.29) | 0.87 (0.60, 1.25) | 0.93 (0.64, 1.35) |
| Q4 | 66 / 1048 | 0.96 (0.68, 1.38) | 0.94 (0.66, 1.35) | 1.01 (0.69, 1.46) |
| Per SD | / | 1.02 (0.89, 1.16) | 1.01 (0.88, 1.15) | 1.03 (0.91, 1.18) |
| **AIP** |  |  |  |  |
| Q1 | 71 / 1048 | 1.00 (Reference) | 1.00 (Reference) | 1.00 (Reference) |
| Q2 | 60 / 1044 | 0.84 (0.59, 1.21) | 0.83 (0.58, 1.20) | 0.89 (0.62, 1.28) |
| Q3 | 61 / 1046 | 0.85 (0.60, 1.22) | 0.81 (0.56, 1.16) | 0.85 (0.59, 1.23) |
| Q4 | 72 / 1047 | 1.14 (0.81, 1.62) | 1.06 (0.74, 1.50) | 1.12 (0.78, 1.61) |
| Per SD | / | 1.04 (0.92, 1.19) | 1.01 (0.89, 1.16) | 1.04 (0.90, 1.18) |
| **TyG-AIP** |  |  |  |  |
| Q1 | 73 / 1047 | 1.00 (Reference) | 1.00 (Reference) | 1.00 (Reference) |
| Q2 | 58 / 1045 | 0.79 (0.55, 1.13) | 0.78 (0.54, 1.12) | 0.83 (0.57, 1.19) |
| Q3 | 61 / 1047 | 0.83 (0.58, 1.18) | 0.78 (0.55, 1.12) | 0.82 (0.57, 1.19) |
| Q4 | 72 / 1046 | 1.11 (0.79, 1.57) | 1.03 (0.72, 1.46) | 1.09 (0.76, 1.56) |
| Per SD |  | 1.05 (0.93, 1.20) | 1.02 (0.90, 1.17) | 1.04 (0.91, 1.19) |

Model 1: adjusted for age, sex,

Model 2 further adjusted for education level, married status, smoking and drinking habits.

Model 3 further adjusted for SBP, obesity, LDL-C.
