# Supplementary material for: Association between triglyceride-glucose-atherogenic index of plasma and cardiovascular disease in middle-aged and older Chinese and American individuals: A cross-sectional analysis of two nationwide cohort datasets
Source: Medicine (Baltimore). 2026 May 8;105(19):e48675. doi: 10.1097/MD.0000000000048675 (PMC13166467; doi:10.1097/MD.0000000000048675)
Supplement: Supplementary file 6 [file medi-105-e48675-s006.docx]

**Table S5.** Stratified analysis for association of AIP with CVD in **CHARLS**

|  | OR (95%CI) | | | |  |
| --- | --- | --- | --- | --- | --- |
|  | Q1 | Q2 | Q3 | Q4 | *P*-interaction |
| Sex |  |  |  |  | 0.77 |
| Male | 1.00 (Reference) | 1.29 (1.03, 1.63) | 1.53 (1.23, 1.92) | 1.94 (1.56, 2.41) |  |
| Female | 1.00 (Reference) | 1.17 (0.91, 1.50) | 1.34 (1.05, 1.72) | 1.64 (1.29, 2.08) |  |
| Marital status |  |  |  |  | 0.55 |
| Live without spouse | 1.00 (Reference) | 1.23 (1.02, 1.48) | 1.41 (1.18, 1.69) | 1.84 (1.55, 2.19) |  |
| Live with spouse | 1.00 (Reference) | 1.38 (0.91, 2.10) | 1.85 (1.24, 2.78) | 1.84 (1.21, 2.80) |  |
| Education attainment |  |  |  |  | 0.73 |
| Middle school or below | 1.00 (Reference) | 1.26 (1.03, 1.53) | 1.52 (1.26, 1.85) | 1.79 (1.48, 2.17) |  |
| High school or above | 1.00 (Reference) | 1.24 (0.90, 1.72) | 1.37 (1.00, 1.88) | 1.93 (1.44, 2.61) |  |
| Tobacco smoking |  |  |  |  | 0.81 |
| Non-smoker | 1.00 (Reference) | 1.24 (1.02, 1.51) | 1.42 (1.18, 1.72) | 1.84 (1.53, 2.21) |  |
| Smoker | 1.00 (Reference) | 1.25 (0.90, 1.74) | 1.55 (1.12, 2.15) | 1.68 (1.23, 2.33) |  |
| Alcohol consumption |  |  |  |  | 0.91 |
| Non-drinker | 1.00 (Reference) | 1.25 (1.02, 1.53) | 1.42 (1.17, 1.73) | 1.78 (1.48, 2.16) |  |
| Drinker | 1.00 (Reference) | 1.09 (0.79, 1.50) | 1.37 (1.00, 1.86) | 1.70 (1.27, 2.28) |  |
| Obesity |  |  |  |  | 0.93 |
| No | 1.00 (Reference) | 1.23 (1.03, 1.47) | 1.43 (1.20, 1.70) | 1.71 (1.44, 2.03) |  |
| Yes | 1.00 (Reference) | 1.14 (0.65, 2.06) | 1.20 (0.71, 2.09) | 1.50 (0.92, 2.57) |  |

Model adjusted for age, sex, education level, married status, smoking and drinking habits, SBP, obesity, LDL-C
