# Supplementary material for: Association between triglyceride-glucose-atherogenic index of plasma and cardiovascular disease in middle-aged and older Chinese and American individuals: A cross-sectional analysis of two nationwide cohort datasets
Source: Medicine (Baltimore). 2026 May 8;105(19):e48675. doi: 10.1097/MD.0000000000048675 (PMC13166467; doi:10.1097/MD.0000000000048675)
Supplement: Supplementary file 7 [file medi-105-e48675-s007.docx]

**Table S6.** Stratified analysis for association of TyG-AIP with CVD in **CHARLS**

|  | OR (95%CI) | | | |  |
| --- | --- | --- | --- | --- | --- |
|  | Q1 | Q2 | Q3 | Q4 | *P*-interaction |
| Sex |  |  |  |  | 0.76 |
| Male | 1.00 (Reference) | 1.28 (1.02, 1.62) | 1.54 (1.24, 1.93) | 1.93 (1.56, 2.40) |  |
| Female | 1.00 (Reference) | 1.13 (0.88, 1.46) | 1.35 (1.06, 1.72) | 1.63 (1.28, 2.06) |  |
| Marital status |  |  |  |  | 0.59 |
| Live without spouse | 1.00 (Reference) | 1.20 (1.00, 1.44) | 1.42 (1.19, 1.70) | 1.82 (1.53, 2.16) |  |
| Live with spouse | 1.00 (Reference) | 1.44 (0.95, 2.19) | 1.86 (1.25, 2.80) | 1.91 (1.26, 2.90) |  |
| Education attainment |  |  |  |  | 0.78 |
| Middle school or below | 1.00 (Reference) | 1.25 (1.02, 1.52) | 1.53 (1.27, 1.86) | 1.79 (1.48, 2.17) |  |
| High school or above | 1.00 (Reference) | 1.19 (0.86, 1.65) | 1.37 (1.01, 1.88) | 1.89 (1.41, 2.55) |  |
| Tobacco smoking |  |  |  |  | 0.92 |
| Non-smoker | 1.00 (Reference) | 1.21 (1.00, 1.48) | 1.43 (1.19, 1.73) | 1.81 (1.51, 2.18) |  |
| Smoker | 1.00 (Reference) | 1.25 (0.90, 1.74) | 1.55 (1.12, 2.14) | 1.73 (1.25, 2.38) |  |
| Alcohol consumption |  |  |  |  | 0.84 |
| Non-drinker | 1.00 (Reference) | 1.23 (1.01, 1.51) | 1.43 (1.18, 1.74) | 1.77 (1.46, 2.14) |  |
| Drinker | 1.00 (Reference) | 1.04 (0.76, 1.44) | 1.37 (1.01, 1.87) | 1.70 (1.27, 2.29) |  |
| Obesity |  |  |  |  | 0.92 |
| No | 1.00 (Reference) | 1.21 (1.01, 1.44) | 1.43 (1.20, 1.70) | 1.71 (1.44, 2.03) |  |
| Yes | 1.00 (Reference) | 1.12 (0.64, 2.03) | 1.20 (0.71, 2.09) | 1.48 (0.90, 2.53) |  |

Model adjusted for age, sex, education level, married status, smoking and drinking habits, SBP, obesity, LDL-C.
