# Supplementary material for: Association between triglyceride-glucose-atherogenic index of plasma and cardiovascular disease in middle-aged and older Chinese and American individuals: A cross-sectional analysis of two nationwide cohort datasets
Source: Medicine (Baltimore). 2026 May 8;105(19):e48675. doi: 10.1097/MD.0000000000048675 (PMC13166467; doi:10.1097/MD.0000000000048675)
Supplement: Supplementary file 8 [file medi-105-e48675-s008.docx]

**Table S7.** Stratified analysis for association of TyG with CVD in **NHANES**

|  | OR (95%CI) | | | |  |
| --- | --- | --- | --- | --- | --- |
|  | Q1 | Q2 | Q3 | Q4 | *P*-interaction |
| Sex |  |  |  |  | 0.63 |
| Male | 1.00 (Reference) | 1.07 (0.77, 1.48) | 1.10 (0.80, 1.53) | 1.53 (1.13, 2.08) |  |
| Female | 1.00 (Reference) | 1.35 (0.96, 1.92) | 1.49 (1.06, 2.11) | 1.90 (1.36, 2.68) |  |
| Marital status |  |  |  |  | 0.43 |
| Live without spouse | 1.00 (Reference) | 1.34 (0.97, 1.85) | 1.31 (0.95, 1.80) | 1.98 (1.46, 2.69) |  |
| Live with spouse | 1.00 (Reference) | 1.06 (0.74, 1.51) | 1.30 (0.91, 1.85) | 1.46 (1.04, 2.05) |  |
| Education attainment |  |  |  |  | 0.36 |
| Middle school or below | 1.00 (Reference) | 1.37 (1.01, 1.88) | 1.52 (1.11, 2.06) | 1.94 (1.45, 2.62) |  |
| High school or above | 1.00 (Reference) | 0.98 (0.67, 1.43) | 1.01 (0.69, 1.45) | 1.44 (1.02, 2.06) |  |
| Tobacco smoking |  |  |  |  | 0.07 |
| Non-smoker | 1.00 (Reference) | 0.85 (0.48, 1.48) | 1.43 (0.88, 2.34) | 1.22 (0.74, 2.03) |  |
| Smoker | 1.00 (Reference) | 1.31 (1.00, 1.70) | 1.21 (0.92, 1.59) | 1.87 (1.45, 2.42) |  |
| Alcohol consumption |  |  |  |  | 0.78 |
| Non-drinker | 1.00 (Reference) | 1.22 (0.88, 1.70) | 1.22 (0.87, 1.70) | 1.57 (1.14, 2.17) |  |
| Drinker | 1.00 (Reference) | 1.15 (0.82, 1.63) | 1.32 (0.94, 1.85) | 1.85 (1.34, 2.56) |  |
| Obesity |  |  |  |  | 0.67 |
| No | 1.00 (Reference) | 1.15 (0.86, 1.53) | 1.23 (0.91, 1.65) | 1.48 (1.09, 2.00) |  |
| Yes | 1.00 (Reference) | 1.33 (0.86, 2.08) | 1.37 (0.91, 2.08) | 1.99 (1.36, 2.97) |  |

Model adjusted for age, sex, education level, married status, smoking and drinking habits, SBP, obesity, LDL-C
