# Supplementary material for: Association between triglyceride-glucose-atherogenic index of plasma and cardiovascular disease in middle-aged and older Chinese and American individuals: A cross-sectional analysis of two nationwide cohort datasets
Source: Medicine (Baltimore). 2026 May 8;105(19):e48675. doi: 10.1097/MD.0000000000048675 (PMC13166467; doi:10.1097/MD.0000000000048675)
Supplement: Supplementary file 9 [file medi-105-e48675-s009.docx]

**Table S8.** Stratified analysis for association of AIP with CVD in **NHANES**

|  | OR (95%CI) | | | |  |
| --- | --- | --- | --- | --- | --- |
|  | Q1 | Q2 | Q3 | Q4 | *P*-interaction |
| Sex |  |  |  |  | 0.92 |
| Male | 1.00 (Reference) | 1.13 (0.79, 1.60) | 1.49 (1.07, 2.09) | 1.61 (1.17, 2.22) |  |
| Female | 1.00 (Reference) | 1.02 (0.73, 1.43) | 1.35 (0.97, 1.87) | 1.65 (1.18, 2.30) |  |
| Marital status |  |  |  |  | 0.47 |
| Live without spouse | 1.00 (Reference) | 1.12 (0.80, 1.55) | 1.43 (1.05, 1.97) | 1.91 (1.42, 2.60) |  |
| Live with spouse | 1.00 (Reference) | 1.06 (0.74, 1.52) | 1.51 (1.07, 2.12) | 1.46 (1.03, 2.06) |  |
| Education attainment |  |  |  |  | 0.07 |
| Middle school or below | 1.00 (Reference) | 1.24 (0.91, 1.69) | 1.41 (1.04, 1.92) | 2.02 (1.51, 2.72) |  |
| High school or above | 1.00 (Reference) | 0.88 (0.60, 1.30) | 1.49 (1.04, 2.12) | 1.29 (0.91, 1.85) |  |
| Tobacco smoking |  |  |  |  | 0.22 |
| Non-smoker | 1.00 (Reference) | 1.30 (0.75, 2.28) | 1.05 (0.62, 1.80) | 1.46 (0.90, 2.42) |  |
| Smoker | 1.00 (Reference) | 1.05 (0.80, 1.37) | 1.55 (1.20, 2.01) | 1.71 (1.32, 2.21) |  |
| Alcohol consumption |  |  |  |  | 0.81 |
| Non-drinker | 1.00 (Reference) | 0.99 (0.70, 1.40) | 1.47 (1.06, 2.04) | 1.56 (1.13, 2.16) |  |
| Drinker | 1.00 (Reference) | 1.11 (0.79, 1.57) | 1.35 (0.98, 1.89) | 1.72 (1.25, 2.37) |  |
| Obesity |  |  |  |  | 0.72 |
| No | 1.00 (Reference) | 1.01 (0.75, 1.35) | 1.32 (0.99, 1.76) | 1.49 (1.11, 1.99) |  |
| Yes | 1.00 (Reference) | 1.27 (0.81, 2.00) | 1.68 (1.12, 2.59) | 1.97 (1.32, 2.99) |  |

Model adjusted for age, sex, education level, married status, smoking and drinking habits, SBP, obesity, LDL-C.
