# Supplementary material for: Association between triglyceride-glucose-atherogenic index of plasma and cardiovascular disease in middle-aged and older Chinese and American individuals: A cross-sectional analysis of two nationwide cohort datasets
Source: Medicine (Baltimore). 2026 May 8;105(19):e48675. doi: 10.1097/MD.0000000000048675 (PMC13166467; doi:10.1097/MD.0000000000048675)
Supplement: Supplementary file 10 [file medi-105-e48675-s010.docx]

**Table S9.** Stratified analysis for association of TyG-AIP with CVD in **NHANES**

|  | OR (95%CI) | | | |  |
| --- | --- | --- | --- | --- | --- |
|  | Q1 | Q2 | Q3 | Q4 | *P*-interaction |
| Sex |  |  |  |  | 0.84 |
| Male | 1.00 (Reference) | 1.14 (0.80, 1.62) | 1.51 (1.08, 2.11) | 1.60 (1.17, 2.22) |  |
| Female | 1.00 (Reference) | 1.01 (0.72, 1.41) | 1.32 (0.95, 1.83) | 1.66 (1.19, 2.31) |  |
| Marital status |  |  |  |  | 0.57 |
| Live without spouse | 1.00 (Reference) | 1.07 (0.77, 1.49) | 1.42 (1.04, 1.95) | 1.87 (1.38, 2.54) |  |
| Live with spouse | 1.00 (Reference) | 1.11 (0.78, 1.59) | 1.51 (1.07, 2.13) | 1.50 (1.06, 2.12) |  |
| Education attainment |  |  |  |  | 0.11 |
| Middle school or below | 1.00 (Reference) | 1.24 (0.91, 1.69) | 1.43 (1.05, 1.94) | 2.00 (1.49, 2.69) |  |
| High school or above | 1.00 (Reference) | 0.88 (0.60, 1.29) | 1.46 (1.02, 2.08) | 1.31 (0.92, 1.88) |  |
| Tobacco smoking |  |  |  |  | 0.20 |
| Non-smoker | 1.00 (Reference) | 1.38 (0.79, 2.42) | 1.10 (0.65, 1.89) | 1.48 (0.91, 2.45) |  |
| Smoker | 1.00 (Reference) | 1.03 (0.79, 1.35) | 1.53 (1.19, 1.98) | 1.70 (1.32, 2.20) |  |
| Alcohol consumption |  |  |  |  | 0.77 |
| Non-drinker | 1.00 (Reference) | 1.00 (0.71, 1.41) | 1.48 (1.07, 2.07) | 1.57 (1.14, 2.18) |  |
| Drinker | 1.00 (Reference) | 1.11 (0.78, 1.56) | 1.33 (0.96, 1.86) | 1.72 (1.25, 2.37) |  |
| Obesity |  |  |  |  | 0.73 |
| No | 1.00 (Reference) | 1.02 (0.77, 1.37) | 1.36 (1.02, 1.81) | 1.46 (1.09, 1.97) |  |
| Yes | 1.00 (Reference) | 1.21 (0.78, 1.91) | 1.59 (1.06, 2.45) | 1.95 (1.32, 2.95) |  |

Model adjusted for age, sex, education level, married status, smoking and drinking habits, SBP, obesity, LDL-C.
